# Expression and anti-inflammatory role of activin receptor-interacting protein 2 in lipopolysaccharide-activated macrophages

**DOI:** 10.1038/s41598-017-10855-4

**Published:** 2017-09-04

**Authors:** Qian Wu, Yan Qi, Na Wu, Chunhui Ma, Wenfang Feng, Xueling Cui, Zhonghui Liu

**Affiliations:** 1 Department of Immunology, College of Basic Medical Sciences, Jilin University, Changchun, China; 2 Department of Genetics, College of Basic Medical Sciences, Jilin University, Changchun, China

## Abstract

The bacterial endotoxin lipopolysaccharide (LPS), a key pathogenic stimulator, can induce the activation of macrophages. Activin receptor-interacting protein 2 (ARIP2), an intracellular signaling protein, has a wide histological distribution, however, whether ARIP2 is involved in regulation of activation of macrophages was not well characterized. Here, by immunocytochemical staining, we found that ARIP2 protein existed in monocyte-macrophage cell line RAW264.7 cells and peritoneal macrophages of mouse, and ARIP2 expression in RAW264.7 cells was up-regulated by LPS. Furthermore, the results revealed that ARIP2 overexpression in the LPS-activated RAW264.7 cells inhibited the productions of IL-1β and TNFα, phagocytic activities and CD14 expression, whereas did not alter expressions of MyD88, TLR2 and TLR4. Additionally, *in vivo* ARIP2 overexpression also reduced the productions of IL-1β and TNFα from the LPS-stimulated peritoneal macrophages of mouse. These data suggest that ARIP2 may play an anti-inflammatory role in macrophages via inhibiting CD14 expression.

## Introduction

Macrophages, as the pivotal innate immune cells, are in the first line of defense against the invasion of pathogens^1^, and play the crucial roles in phagocytosis, sterilization, removal body damage and senescence cells, tissue development and repair, and antigen information transmission^2–4^. Macrophages are also the major effector cells in the inflammatory response, and excess inflammation can lead to tissue damage if the pro-inflammatory response is not terminated at the appropriate time^5–9^. The biological activities of macrophages are regulated by a variety of factors, for example, the bacterial endotoxin lipopolysaccharide (LPS) as inflammatory stimuli can induce activation of macrophages, but activin A inhibits activities of LPS-activated macrophages^10, 11^.

Activin A as an anti-inflammatory cytokine is involved in many physiological processes of the body, such as cell migration and proliferation, bone remodeling and embryo development, *et al*.^12–15^. Activin signaling is transduced via heteromeric complexes composed of two different serine/threonine kinase receptors, termed type I and type II (ActRI and ActRII). Activin A first binds to the type II receptor, the type II receptor transphosphorylates and activates the type I receptor kinase at the membrane region. Then, the type I receptor cytoplasmic domain interacts with intracellular signaling molecules, Smads, which regulate transcription of target genes^16, 17^. Activin recptor-interacting protein 2 (ARIP2) expressed in a variety of tissues is the upstream signal regulatory protein of Smads, and can inhibit production of collagen type IV in mouse Hepa1-6 cells^17–21^. However, it is not well characterized whether ARIP2 is involved in regulation of activation of macrophages.

In the present study, we confirmed that ARIP2 protein existed in monocyte-macrophage cell line RAW264.7 cells and peritoneal macrophages of mouse, and ARIP2 expression was up-regulated by LPS. Furthermore, it was investigated that ARIP2 overexpression *in vitro* in RAW264.7 and *in vivo* in peritoneal macrophages of mouse influenced the activities of LPS-activated macrophages.

## Results

### Expression of ARIP2 protein in mouse macrophages

**Figure 1:**
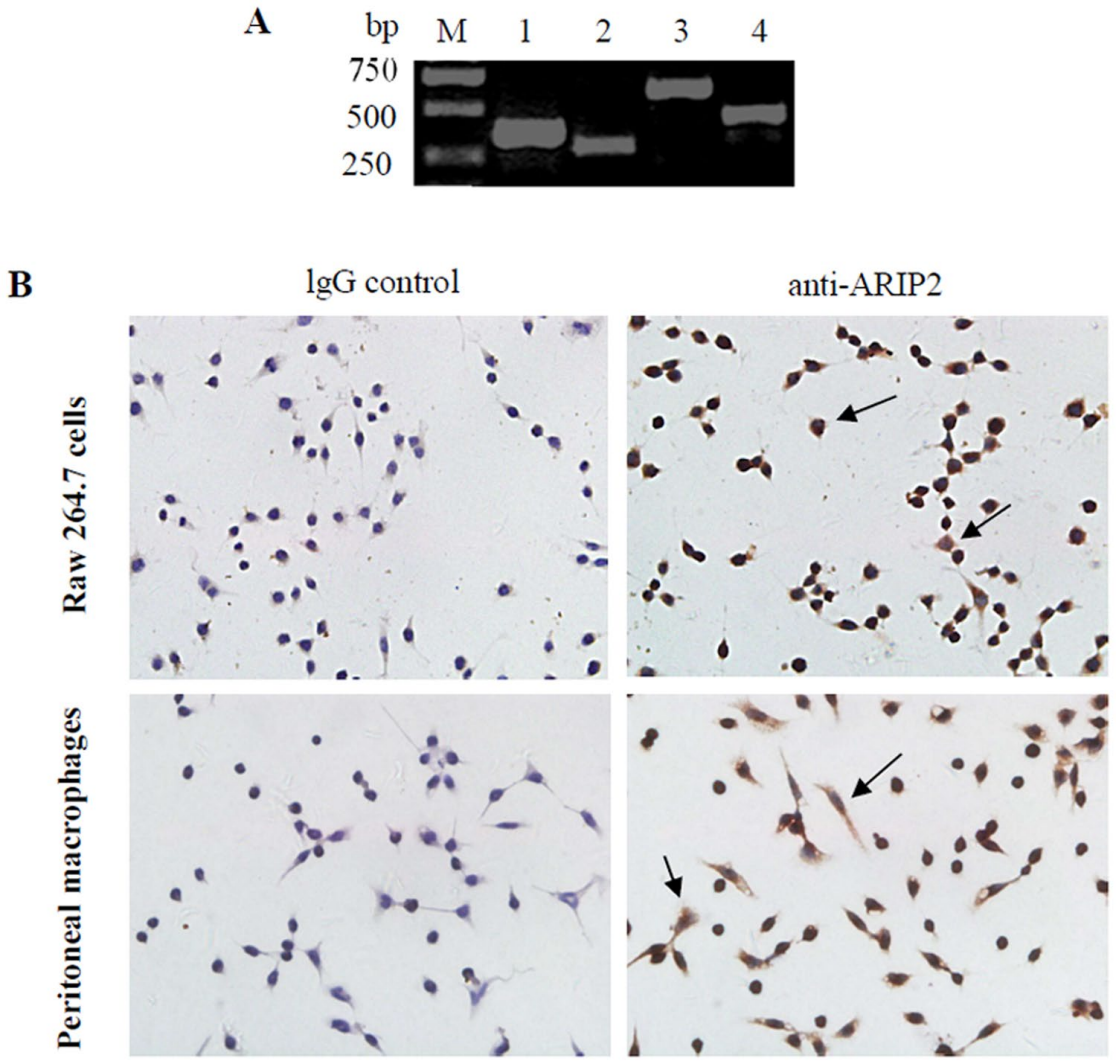
Expression of ARIP2 in mouse macrophages. (**A**) Expressions of ARIP2, ActRIIA and Smad3 mRNA in monocyte-macrophage cell line Raw264.7 cells were examined by RT-PCR. M, molecular weight (bp); lane 1, GAPDH (371 bp); lane 2, ActRIIA (294 bp); lane 3, Smad3 (574 bp); lane 4, ARIP2 (444 bp). Full-length gels are presented in Supplementary Figure 1. (**B**) Expression of mature ARIP2 protein in Raw264.7 cells and peritoneal macrophages of mouse were detected by immunocytochemical staining with rabbit anti-ARIP2 antibody, and control staining was performed with normal rabbit IgG. Arrows represented ARIP2 positive cells (200×).

To assess the expression of ARIP2 in macrophages, ARIP2 mRNA was first examined by RT-PCR. The results showed that the expressions of ARIP2, ActRIIA and Smad3 mRNA were all detectable in mouse monocyte-macrophage cell line RAW264.7 cells (Fig. 1A). Furthermore, the immunocytochemical staining showed that ARIP2 protein was also expressed in RAW264.7 cells and peritoneal macrophages of mouse (Fig. 1B), indic-ating that ARIP2 protein exists in mouse macrophages.

### Effect of LPS on expression of ARIP2 in macrophages

**Figure 2:**
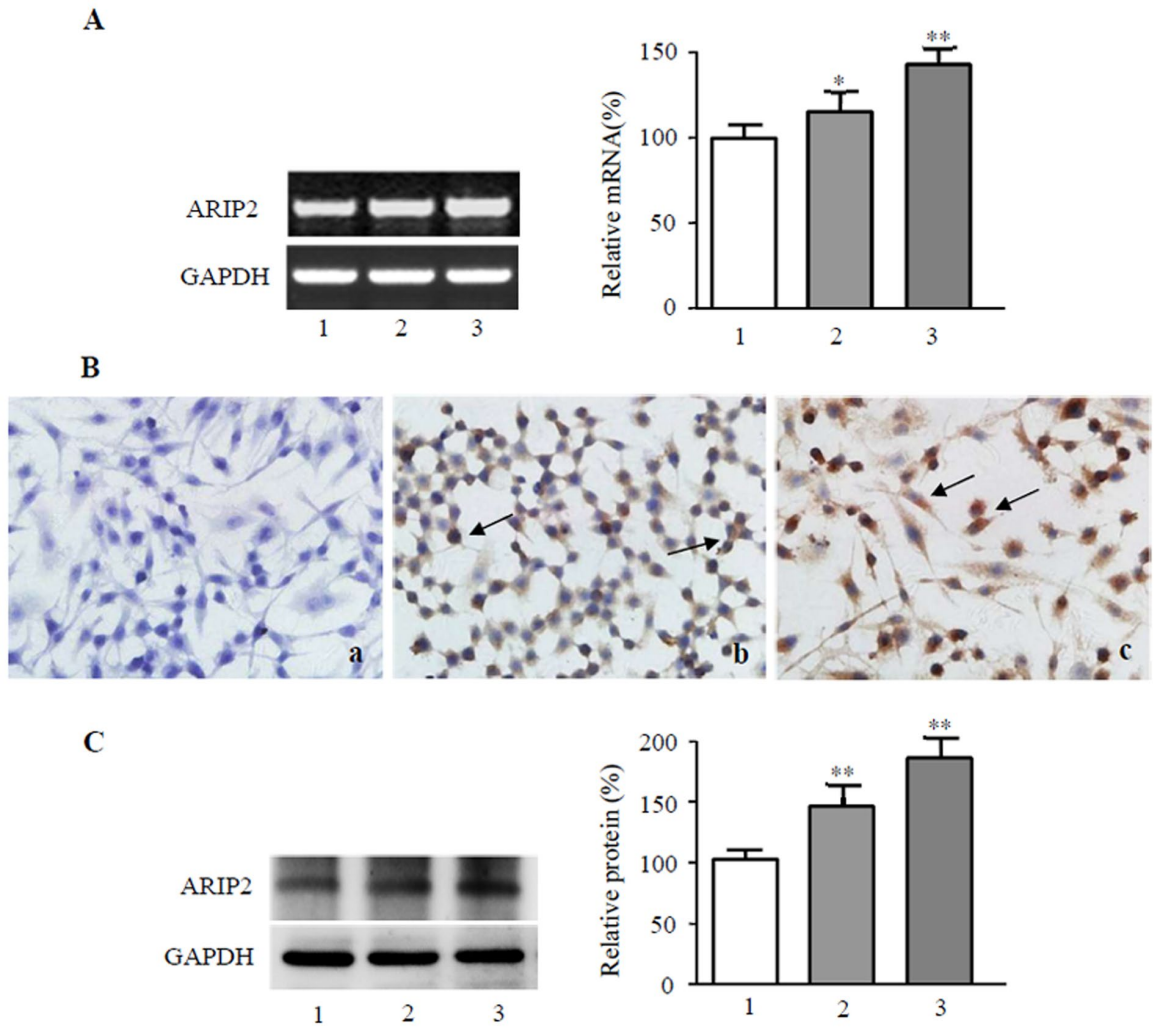
Effects of LPS on expression of ARIP2 in Raw264.7 cells. (**A**) ARIP2 mRNA expression was examined by RT-PCR in Raw264.7 cells treated with LPS for 12 h. Lane 1, Control; Lane 2, LPS 200 ng/ml; Lane 3, LPS 500 ng/ml. The graph represented the relative levels of ARIP2 mRNA in three separate experiments, and the mRNA levels of control group were adjusted to 100%. *P < 0.05, **P < 0.01, compared with control. (**B**) Expression of mature ARIP2 protein in Raw264.7 cells were detected by immunocytochemical staining with normal rabbit IgG as control staining (a) and with anti-ARIP2 antibody 12 h after untreated (b) or treated with LPS 500 ng/ml (c). Arrows represented ARIP2 positive cells (200×). (**C**) Levels of ARIP2 protein expression were examined by Western blotting in Raw264.7 cells treated with LPS for 12 h. Lane 1, Control; Lane 2, LPS 200 ng/ml; Lane 3, LPS 500 ng/ml. The graph represented the relative levels of ARIP2 protein in three separate experiments, and the protein levels of control group were adjusted to 100%. **P < 0.01, compared with control. Full-length blots/gels are presented in Supplementary Figure 2.

To determine whether ARIP2 is engaged in the activation of macrophages, ARIP2 expression was examined in LPS-activated mouse monocyte-macrophage cell line RAW264.7 cells. The results showed that LPS p-romoted the expression of ARIP2 mRNA (Fig. 2A), and also enhanced the expression of mature ARIP2 protein in RAW264.7 cells (Fig. 2B). In addition, by Western blotting, our data further confirmed that LPS increased the levels of ARIP2 protein expression in RAW264.7 cells (Fig. 2C). These results indicated that the expression of ARIP2 was up-regulated by LPS, suggesting that ARIP2 may be involved in the process of macrophage activation induced by LPS.

### Effects of ARIP2 overexpression on cytokines production from RAW264.7 cells

**Figure 3:**
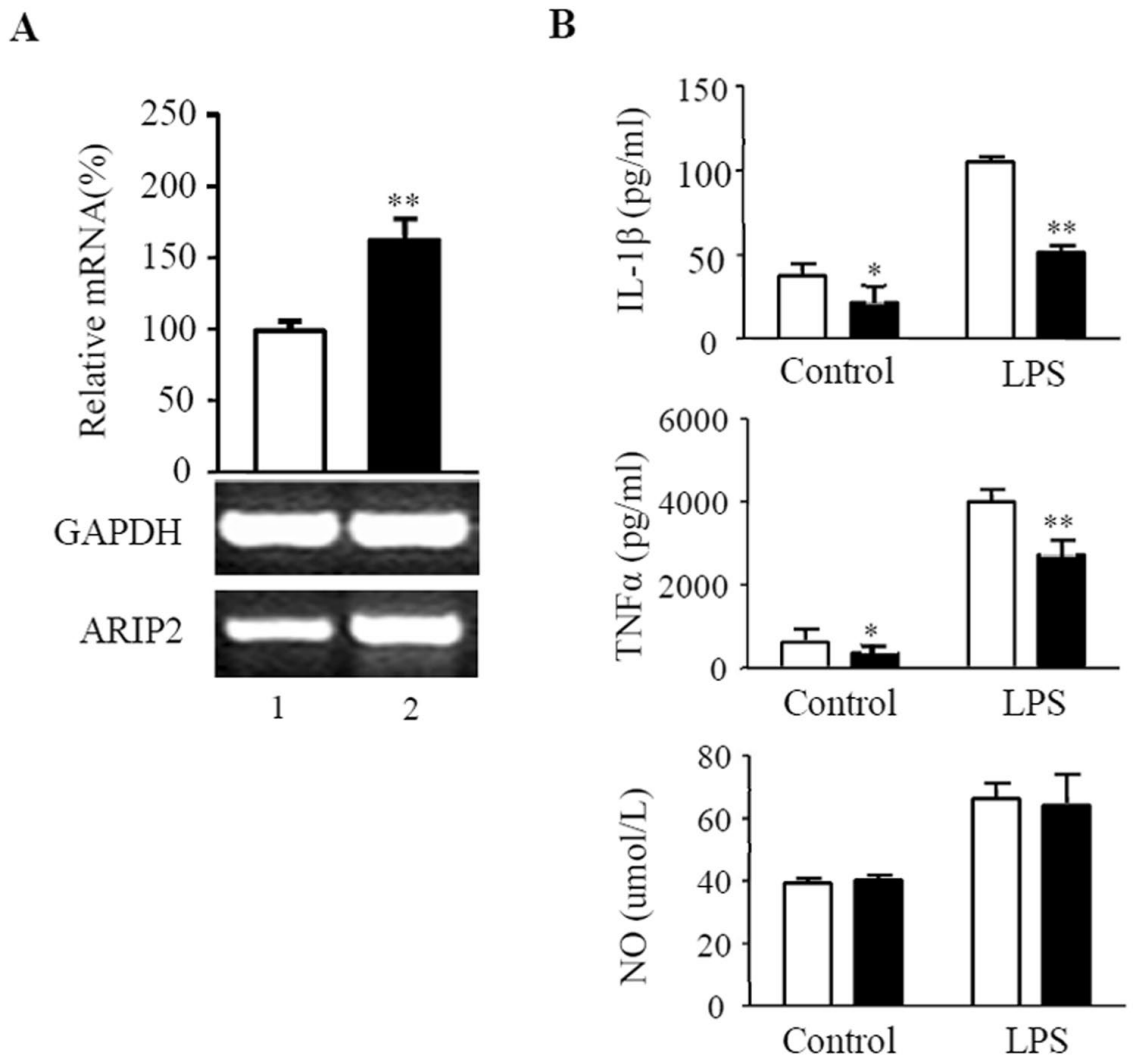
Effects of ARIP2 overexpression on production of IL-1β, TNFα and NO in Raw264.7 cells. (**A**) Expression of ARIP2 mRNA was detected by RT-PCR in Raw264.7 cells transfected with control pcDNA3 empty plasmids (Lane 1) and pcDNA3-ARIP2 expressing plasmids (Lane 2). The graph represented the relative levels of ARIP2 mRNA in three separate experiments, and the mRNA levels of control group were adjusted to 100%. **P < 0.01, compared with control group. Full-length gels are presented in Supplementary Figure 3. (**B**) The levels of IL-1β and TNFα were detected by ELISA in the supernatants of the cultured pcDNA3-ARIP2 expressing plasmid (ARIP2) and pcDNA3 empty plasmid (PC)-transfected Raw264.7 cells treated with culture medium (Control) or LPS 500ng/ml for 12 h, and the levels of NO were measured by Griess. □, PC group; ■ ARIP2 group. *P < 0.05, **P < 0.01, compared with PC group.

Here, the results revealed the apparently higher levels of ARIP2 mRNA expression in RAW264.7 cells transfected with pcDNA3-ARIP2 plasmids than those in RAW264.7 cells transfected with control plasmids (Fig. 3A), indicating that the ARIP2 was successfully overexpressed in RAW264.7 cells. Furthermore, the levels of IL-1β, TNFα and NO in the supernatants of cultured RAW264.7 cells were determined. The results showed that ARIP2 overexpression reduced the production of IL-1β and TNFα from RAW264.7 cells in the presence or absence of LPS, but had no significant effect on NO secretion (Fig. 3B), suggesting that ARIP2 overexpression in macrophages might play an anti-inflammatory role through inhibiting pro-inflammatory cytokines IL-1β and TNFα production.

### Inhibition of phagocytosis in RAW264.7 cells by ARIP2 overexpression

**Figure 4:**
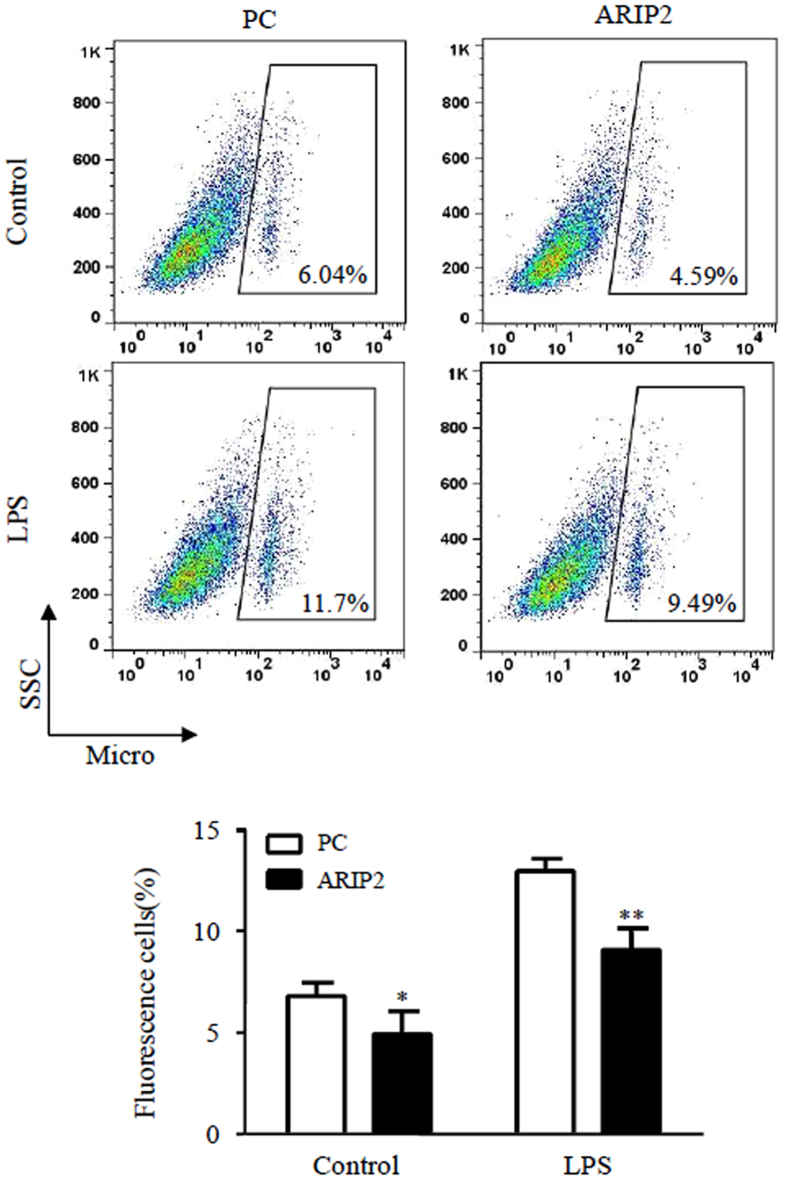
Inhibitory effect of ARIP2 overexpression on phagocytosis of LPS-activated Raw264.7 cells. Phagocytic activities to red fluorescent microspheres were examined by flow cytometry in control empty plasmid (PC) or ARIP2 expressing plasmid (ARIP2)-transfected Raw264.7 cells treated with LPS or culture medium (Control) for 12 h. The graph showed the phagocytic activities of Raw264.7 cells in three separate experiments. *P < 0.05, **P < 0.01, compared with PC group.

Macrophages possess the ability to phagocytose and kill pathogens^22, 23^. In this study, flow cytometry was used to examine the effect of ARIP2 overexpression on phagocytosis of RAW264.7 cells. The result showed that there were lower phagocytic activities to red fluorescent microspheres in ARIP2-overexpressing RAW264.7 cells than those in control plasmid-transfected RAW264.7 cells in the presence or absence of LPS (Fig. 4).

### Effect of ARIP2 overexpression on CD14 and TLR2, 4 expressions

**Figure 5:**
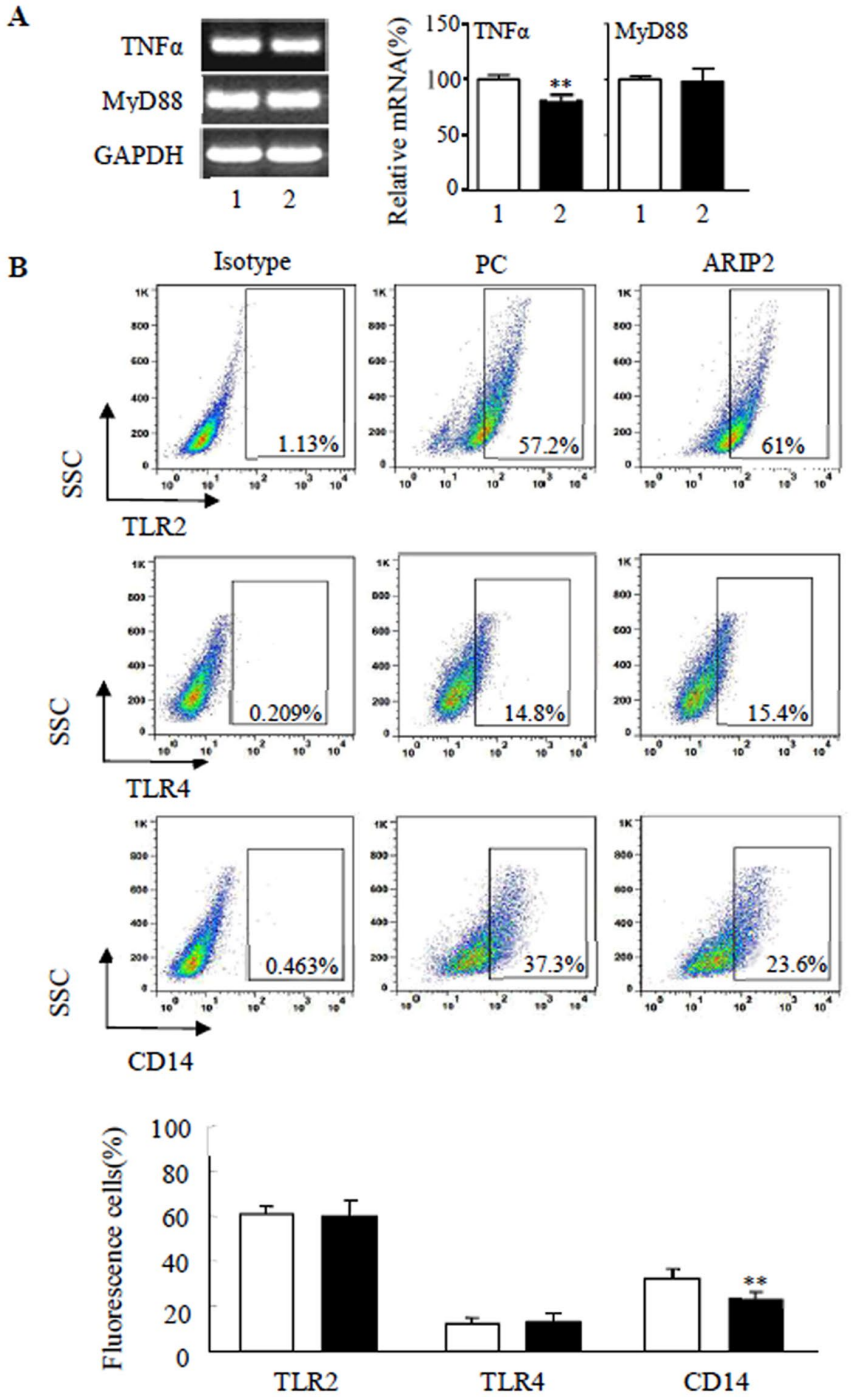
Effects of ARIP2 overexpression on MyD88, TLR2, TLR4 and CD14 expressions in Raw264.7 cells. (**A**) TNFα and MyD88 mRNA expressions were examined by RT-PCR in Raw264.7 cells transfected with pcDNA3 empty plasmid (Lane 1) or pcDNA3-ARIP2 expressing plasmid (Lane 2). The graph represented the relative levels of mRNA in three separate experiments, and the mRNA levels of PC control group were adjusted to 100%. **P < 0.01, compared with PC group. Full-length gels are presented in Supplementary Figure 4. (**B**) Expression of TLR2, TLR4 and CD14 on macrophages was detected by flow cytometry. The graph showed the TLR2, TLR4 and CD14 expressions on Raw264.7 cells in three independent experiments. □, PC group; ■ ARIP2 group. **P < 0.01, compared with PC group.

LPS stimulates macrophages to produce inflammatory mediators, which is related to TLR2, 4 and CD14 molecules on cell surface^24–26^, and LPS-CD14/TLR4 complex conducts signals through intracellular MyD88 and other molecules^27–30^. Here, we found that ARIP2 overexpression inhibited the expression of TNFα mRNA in RAW264.7 cells, but had no obvious effect on expression of MyD88 mRNA (Fig. 5A). The flow cytometry analysis indicated that ARIP2 overexpression did not induce the significant changes of TLR2 and TLR4 expression on RAW264.7 cells, compared with control group. Whereas CD14 expression was down-regulated significantly on ARIP2-overexpressing RAW264.7 cells, compared with that on control RAW264.7 cells (Fig. 5B), suggesting that ARIP2 may inhibit pro-inflammatory cytokines via attenuating the expression of CD14 on the surface of macrophages.

### Inhibitory effects of *in vivo* ARIP2 overexpression on production of inflammatory cytokines of peritoneal macrophages of mouse

**Figure 6:**
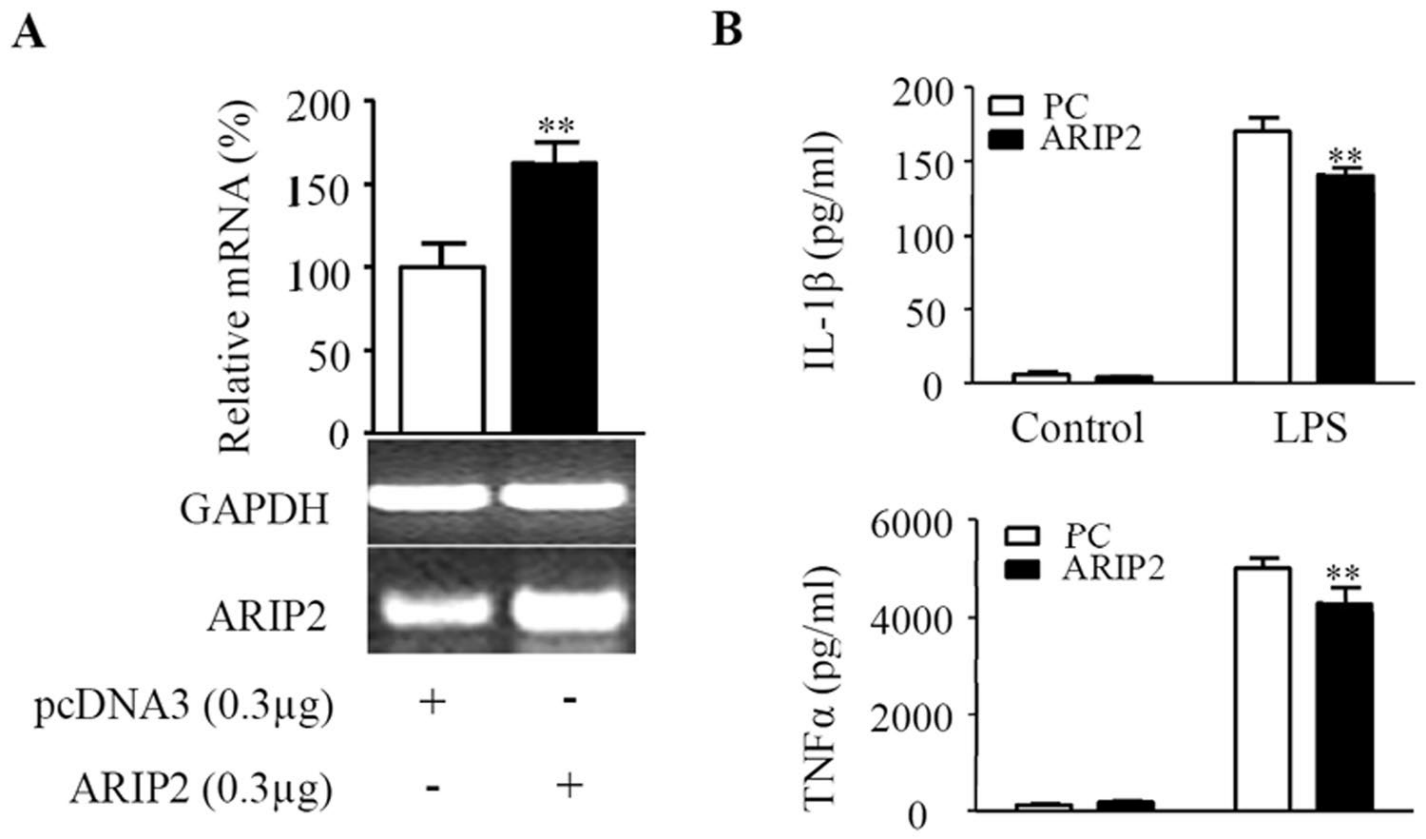
Inhibitory effects of *in vivo* ARIP2 overexpression on production of inflammatory cytokines of mouse peritoneal macrophages. (**A**) Expression of ARIP2 mRNA was detected by RT-PCR in mouse peritoneal macrophages transfected *in vivo* with pcDNA3 empty plasmid or pcDNA3-ARIP2 expressing plasmid (ARIP2). The graph represented the relative levels of mRNA in three separate experiments, and the mRNA levels of pcDNA3 control group were adjusted to 100%. **P < 0.01, compared with control group. Full-length gels are presented in Supplementary Figure 5. (**B**) Levels of IL-1β and TNFα were detected by ELISA in the supernatants of the cultured *in vivo* pcDNA3 (PC) and ARIP2 expressing plasmid-transfected mouse peritoneal macrophages, respectively, untreated (Control) or treated with LPS 500 ng/ml. **P < 0.01, compared with PC group.

To further verify the anti-inflammatory role of ARIP2 in macrophages, peritoneal macrophages of mouse were transfected *in vivo* with pcDNA3-ARIP2 expressing plasmid. The results showed that the level of ARIP2 mRNA expression was exactly higher in ARIP2-overexpressing mouse peritoneal macrophages than those in the control plasmid group (Fig. 6A), indicating that the ARIP2 was successfully overexpressed *in vivo* in mouse peritoneal macrophages transfected with pcDNA3-ARIP2 plasmids. Furthermore, we found that ARIP2 overexpression significantly inhibited productions of IL-1β and TNFα in LPS-activated mouse peritoneal macrophages (Fig. 6B), indicating that *in vivo* ARIP2 overexpression also had anti-inflammatory role in LPS-activated macrophages via reducing the release of inflammatory cytokines.

## Discussion

Macrophages are not only important innate immune cells in body defense system, but also the main cells that are involved in inflammatory response, for example, macrophages participate in acute phase response in inflammatory diseases via secreting pro-inflammatory cytokines TNFα, IL-1β and IL-6, *et al*.^31–34^. As a major component of Gram-negative bacterial cells wall and the main molecular basis of pathogenesis, LPS can induce the activation and maturation of monocytes-macrophages primarily through binding the CD14/TLR4/MD2 receptor complex, resulting in the release of large number of inflammatory mediators, such as NO, TNFα and IL-1β, etc.^28, 30, 35^. If the activated macrophages sustain the release of excessive inflammatory mediators, it can lead to excessive or uncontrolled inflammatory responses, causing damage to the body^36, 37^. In this study, the focus is to investigate the negative regulation of the activated macrophages.

ARIPs belong to activin signal transduction proteins, localized at upstream of Smad signal cascade. ARIP1 has two WW and five PDZ-like domains, interacting with ActRII through the fifth PDZ domain, and inhibits activin signaling transduction^38^. ARIP2 only has one PDZ domain, and binds ActRII through this PDZ domain. Furthermore, ARIP2 can also interact with RalBP1 (Ral-binding protein 1) through its C-terminal peptide to mediate activin receptor endocytosis, and ARIP2 overexpression in mouse hepatoma Hepa1-6 cells can inhibit production of collagen type IV^17, 19^. However, it is still unclear whether LPS can affect the expression of ARIP2 protein in macrophages and ARIP2 is involved in the negative regulation of LPS-activated macrophages.

Previous our studies have reported that ARIP2 widely distributed in the heart, lung, kidney, pancreas, brain and other tissues^18–21^. Therefore, in order to identify the relationship of ARIP2 expression and LPS response in macrophages, we first detected the expression of ARIP2 in mouse peritoneal macrophages and monocyte-macrophage cell line RAW264.7 cells. RT-PCR and immunocytochemical staining showed that ARIP2 was expressed in mouse peritoneal macrophages and RAW264.7 cells. Furthermore, our data showed that LPS up-regulated ARIP2 expression in RAW264.7 cells, suggesting that ARIP2 as activin signaling protein may be also involved in the process of macrophages activation induced by LPS.

As essential innate immune cells, macrophages clear antigens by phagocytosis, pinocytosis, etc. In addition macrophages are involved in inflammatory responses through the release of inflammatory mediators NO, TNFα and IL-1β^36, 37^. Here, we further examined the biological activities of ARIP2 in macrophages. The data indicated that *in vitro* ARIP2 overexpression reduced the production of IL-1β and TNFα in RAW264.7 cells treated with LPS, but had no significant effect on NO secretion. We also found that ARIP2 overexpression inhibited the phagocytosis of LPS-activated RAW264.7 cells significantly. Similar results were found in which *in vivo* ARIP2 overexpression also inhibited IL-1β and TNFα production in LPS-activated mouse peritoneal macrophages. These data indicated that ARIP2 expressed in macrophages might play an anti-inflammatory role via down-regulating IL-1β and TNFα secretion and phagocytosis.

Macrophage activation and synthesis or secretion of cytokines are related to TLRs. TLR2 recognizes lipoteichoic acids and other compounds, and TLR4 interacts with LPS. As a TLR4 agonist, LPS binds to CD14 and TLR4 complexes, and then activates macrophages through the MyD88-dependent and MyD88-independent pathway^28–30^. Therefore, in order to elucidate the anti-inflammatory mechanism of ARIP2 overexpression in LPS-activated macrophages, we further examined the expression of MyD88, TLR2, 4 and CD14. The results showed that ARIP2 overexpression significantly down-regulated the expression of TNFα mRNA in RAW264.7 cells, but did not alter the expression of MyD88 mRNA. We also found that ARIP2 overexpression had no appreciable effect on the expression of TLR2 and TLR4 on RAW264.7 cells, but significantly inhibited the expression of CD14. These results suggested that ARIP2 may reduce the binding of LPS to CD14/TLR4 complex via down-regulating the expression of CD14, leading to inhibition of macrophage activation.

In summary, these data indicate that ARIP2 protein is expressed in macrophages, and its expression can be regulated by LPS. The increased ARIP2 in the LPS-activated macrophages may play an anti-inflammatory role via inhibiting the production of pro-inflammatory cytokines by feedback regulation, which is related to the inhibition of CD14 expression on macrophages.

## Materials and Methods

### Cell line

Mouse monocyte-macrophage cell line RAW264.7 cells (American Type Culture Collec-tion, ATCC, Rockville, MD) were grown in high glucose DMEM medium supplemented with 10% fetal bovine serum (FBS).

### Animals

Specific pathogen free (SPF) male Balbc mice (6 weeks of age, 18–20 g) were purchased from Beijing Huafu-kang Biotechnology Co., Lt d (Bejing, China). Mice were housed and bred in accordance with the Institutional Animal Care and Use Committee of the Jilin University, and all experimental protocols were approved by Jilin University institutional and licensing committee.

### Reagents

LPS from Escherichia coli 0111:B4 was obtained from Sigma (St. Louis, MO, USA). Reverse transcription-PCR (RT-PCR) kit was purchased from Takara Biotechnology Co (Takara, Dalian, China). FluoSpheres® carboxy-latemodified red fluorescent microspheres (1μm diameter) were provided by Invitrogen (California, CA, USA). Enzyme-linked immunosorbent assay (ELISA) kits for IL-1β and TNFα and monoclonal antibodies against mouse Toll-like receptor 2, 4 (TLR2, TLR4) and CD14 were obtained from eBioscince (San Diego, USA). Mouse anti-GAPDH polyclonal antibody was purchased from Sungene Biotech Co., Ltd (Tianjin, China). The pcDNA3-ARIP2 plasmids were constructed by our laboratory^21^.

### Immunocytochemistry staining

RAW264.7 cells and peritoneal macrophages of mouse were seeded into 96-well plates at a density of 4 × 10^4^ cells per well, respectively, and incubated in 10% FBS-high glucose DMEM medium for 24 h at 37 °C under 5% CO_2_. The cells were fixed with 4% paraformaldehyde and incubated in 3% H_2_O_2_ at room temperature (RT) for 10 min. Non-specific reactivity was blocked by a pre-incubation with 2% bovine serum albumin (BSA) for 30 min at RT. The cells were incubated in rabbit anti-mouse ARIP2 antibody (1:400) overnight at 4 °C^21^, and then processed with biotinylated secondary antibodies for 10 min at RT. The cells were further treated with streptomycete-horse-radish peroxidase for 10 min at RT. Immunoreactive products were visualized by 0.03% H_2_O_2_ and 0.05% diaminobenzidine (DAB). The cells were counterstained by hematoxylin counter stain and images captured on a Nikon Eclipse E400 light microscope (Nikon Corporation, Tokyo, Japan). In order to control staining, the cells were incubated with normal rabbit IgG instead of anti-ARIP2 antibody.

### RT-PCR

Total RNA was extracted from RAW264.7 cells using TRIzol reagent (Takara, Dalian, China) according to manufacturer’s instructions and cDNA synthesis was carried out using PrimeScript^®^ 1st Strand cDNA Synthesis Kit (Takara). PCR was performed with target gene-specific primer listed in Table 1. PCR products were separated by electrophoresis on 1.5% agarose, and stained with ethidium bromide. The specific bands were analyzed using IMAGEMASTER VDS (Pharmacia Biotech Company, Uppsala, Sweden).

**Table 1:** Primer sequences were used in RT-PCR.

Gene	Primer	Sequence (5′-3′)	Fragment size(bp)	Tm(°C)
GAPDH	F	GATTGTTGCCATCAACGACC	371	56
	R	GTGCAGGATGCATTGCTGAC		
ActRIIA	F	ATTGGCCAGCATCCATCTCTTG	294	55
	R	GCCACCATCATAGACTAGATTC		
Smad3	F	CCAGCACACAATAACTTGGA	574	58
	R	AGACACACTGGAACAGCGGA		
ARIP2	F	GTCAGCCGTATCAAAGAGGATG	444	56
	R	CTTGTGGCAATACTTCTCTGGTG		
IL-1β	F	ATGGCAACTGTTCCTGAACTC	819	56
	R	GCCCATACTTTAGGAAGACA		
TNFα	F	CATGAGCACAGAAAGCATGATCCG	121	55
	R	AAGCAGGAATGAGAAGAGGCTGAG		
MyD88	F	ATGGTGGTGGTTGTTTCTGACGATT	169	57
	R	CGCATATAGTGATGAACCGCAGGAT		

### Western blotting

Raw264.7 cells were lysed using M-PER® Mammalian Protein Extraction Reagent (Thermo Scientific) containing Halt^TM^ Protease Inhibitors (Thermo Scientific) and centrifuged at 12,000 × g at 4 °C for 30 min, and then the proteins were quantified using the Peirce^TM^ BCA protein assay kit (Thermo Scientific) following the manufacturer’s instruction. 30 μg of proteins were separated by electrophoresis with 12% SDS-PAGE gel, and transferred onto a polyvinylidene difluoride membrane. The membranes were blocked for 1 h and then exposed to an antibody against ARIP2 (1:500) or GAPDH (1:3000, Sungene Biotech) at 4 °C overnight in blocking buffer. The membranes were then probed with a horseradish peroxidase-conjugated second antibody. Finally, the labeled proteins were detected by chemiluminescence (ECLPlus; Amersham Pharmacia Biotech).

### ARIP2 overexpression *in vitro* in RAW264.7 cells

RAW264.7 cells in logarithmic growth phase were seeded into 12-well cell culture plates at a density of 4 × 10^5^ cells/ml, and the cells were transfected with pcDNA3 empty plasmids and pcDNA3-ARIP2 expressing plasmids, respectively, by using the X-tremeGENE HP transfection reagent (Roche) according to the manufacturer’s protocol for 12 h. The transfected RAW264.7 cells were incubated in 2% FBS-DMEM medium in the presence or absence of LPS (500 ng/ml) for 12 h. The supernatants of the cultured cells were collected for ELISA and the cell pellets were used for RT-PCR analysis.

### Detection of IL-1β and TNFα

The supernatants of the cultured RAW264.7 cells were collected, and levels of interleukin-1β (IL-1β) and tumour necrosis factor-α (TNF-α) were examined by ELISA kit according to the manufacturer’s protocol (eBioscience).

### Assay of phagocytosis

RAW264.7 cells were transfected with pcDNA3 empty plasmids and pcDNA3-ARIP2 expressing plasmids, respectively, and then incubated in 2% FBS-DMEM medium in the presence or absence of LPS (500 ng/ml) for 12 h, followed by incubating with 1 × 10^7^ red fluorescent microspheres/well for 1 h, rinsed with pH 7.4, 0.01 mol/L phosphate-buffered saline (PBS). Phagocytosis ratio was performed by flow cytometry (New Jersey, USA, BD Calibur).

### Flow cytometry

Raw264.7 cells were incubated in FITC-conjugated anti-mouse TLR2, and PE-conjugated anti-mouse TLR4 or CD14 antibodies for 30 min at RT, respectively, meanwhile isotype IgG of each antibody was incubated as control. The labeled cells were analyzed by flow cytometry (BD Calibur). The data were collected and analyzed with FlowJo 7.6 to assess the percentage of fluorescence positive cells.

### ARIP2 overexpression *in vivo* in mouse peritoneal macrophages

Mice were randomly divided into pcDNA3 empty plasmid group and pcDNA3-ARIP2 expressing plasmid group after 7 days of adaptive feeding, and treated *in vivo* with ARIP2-expressing plasmid pcDNA-ARIP2 and control empty plasmid pcDNA3, respectively. Briefly, the pcDNA-ARIP2 plasmids (3 μg/mouse) or control pcDNA3 plasmids (3 μg/mouse) were enfolded by X-tremeGENE HP and then injected into mice via intraperitoneal injection. 12 h post-injection of plasmids, the mice peritoneal macrophages were isolated and cultured as previously described^24^.

### Statistical analysis

The data were expressed as mean ± standard deviation (SD), and statistical evaluation was performed by statistical software SPSS 17.0. Differences of P < 0.05 were considered statistically significant.

## Electronic supplementary material

Supplementary Information of full-length gels
