# Supplementary material for: Expression and anti-inflammatory role of activin receptor-interacting protein 2 in lipopolysaccharide-activated macrophages
Source: Sci Rep. 2017 Sep 4;7:10306. doi: 10.1038/s41598-017-10855-4 (PMC5583376; doi:10.1038/s41598-017-10855-4)

Supplementary Information

Expression and anti-inflammatory role of activin receptor-interacting protein 2 in lipopolysaccharide-activated macrophages

Qian Wu[1], Yan Qi[1], Na Wu[1], Chunhui Ma[1], Wenfang Feng[2], Xueling Cui[2], Zhonghui Liu[2]

[1]Department of Immunology, College of Basic Medical Sciences, Jilin University, Changchun, China. [2]Department of Genetics, College of Basic Medical Sciences, Jilin University, Changchun, China

Correspondence and requests for materials should be addressed to Z.L. (email: liuzh@jlu.edu.cn) or X.C. (email: cxl@jlu.edu.cn)

Running title: Anti-inflammatory role of ARIP2 in macrophages

Figure 1. Full-length gels for ARIP2, ActRIIA and Smad3 mRNA in Raw264.7 cells examined by RT-PCR in manuscript Figure 1A. M, molecular weight (bp); lane1, GAPDH; lane2, ActRIIA; lane3, Smad3; lane4, ARIP2.

A

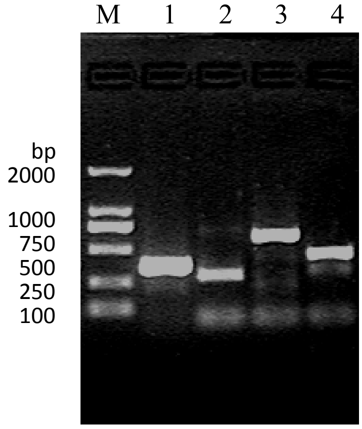

Figure 2. Full-length gels for ARIP2 expression in Raw264.7 cells. (A) Full-length gels for ARIP2 mRNA in Raw264.7 cells treated with LPS in manuscript Figure 2A. Lane 1, Control; Lane 2, LPS 200 ng/ml; Lane 3, LPS 500 ng/ml. (B) Full-length gels for ARIP2 protein in Raw264.7 cells treated with LPS in manuscript Figure 2C. The membrane of full-length gel (a) was cut into (b) size according to colorful molecular marker of protein, and then the (b) size membrane was probed with anti-ARIP2 antibody and the labeled proteins were detected by chemiluminescence (c). M, Colorful molecular marker of protein. Lane 1, Control; Lane 2, LPS 200 ng/ml; Lane 3, LPS 500 ng/ml.

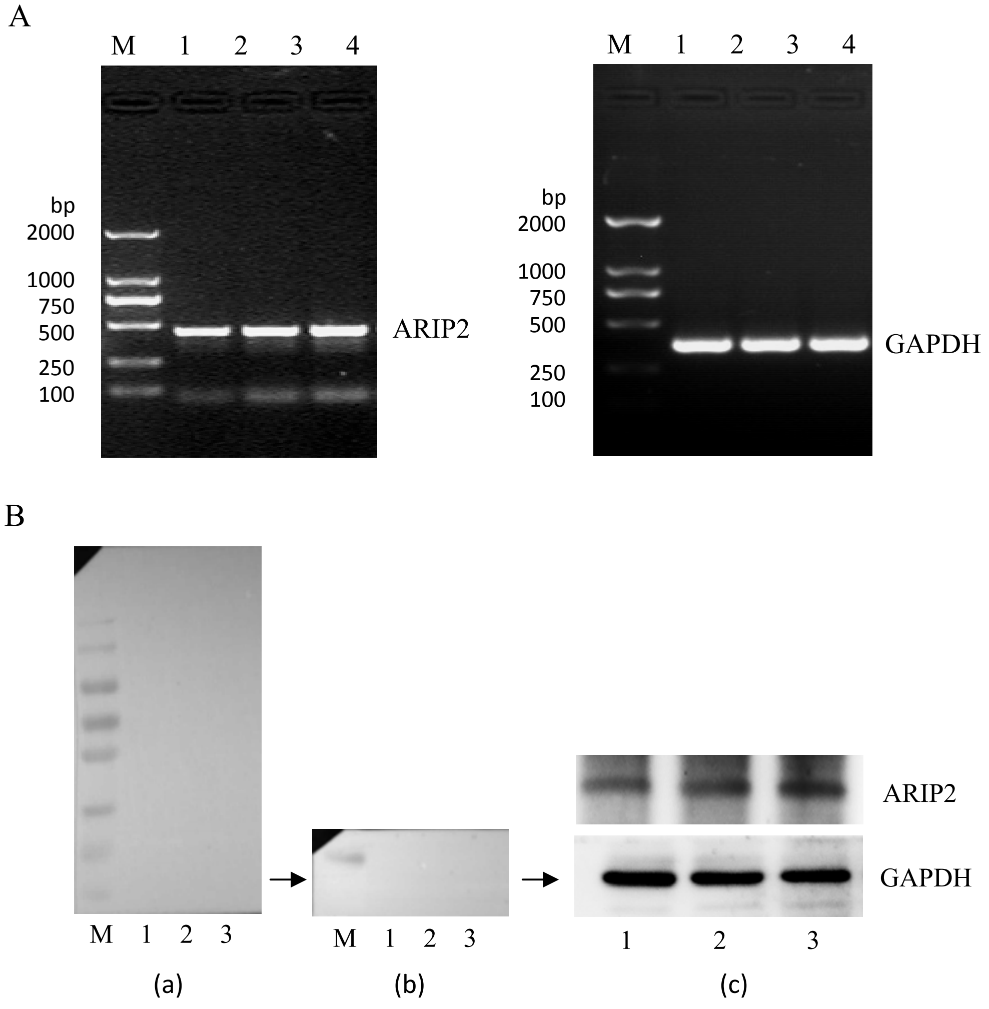

Figure 3. Full-length gels for ARIP2 mRNA in Raw264.7 cells examined by RT-PCR in manuscript Figure 3A. M, molecular weight (bp); lane1, control pcDNA3 empty plasmids; lane2, pcDNA3-ARIP2 expressing plasmids.

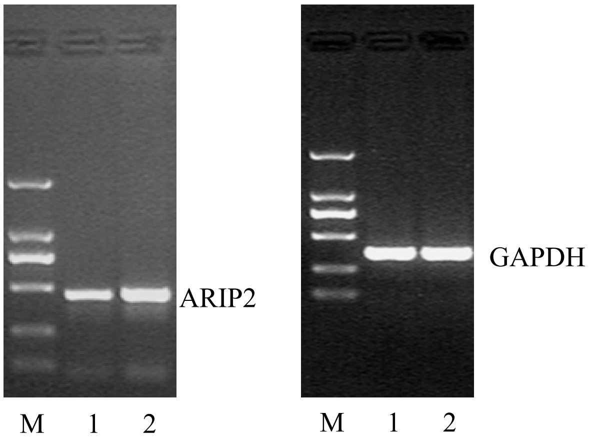

Figure 4. Full-length gels for TNFα and MyD88 mRNA in Raw264.7 cells examined by RT-PCR in manuscript Figure 5A. M, molecular weight (bp); lane1, control pcDNA3 empty plasmids; lane2, pcDNA3-ARIP2 expressing plasmids.

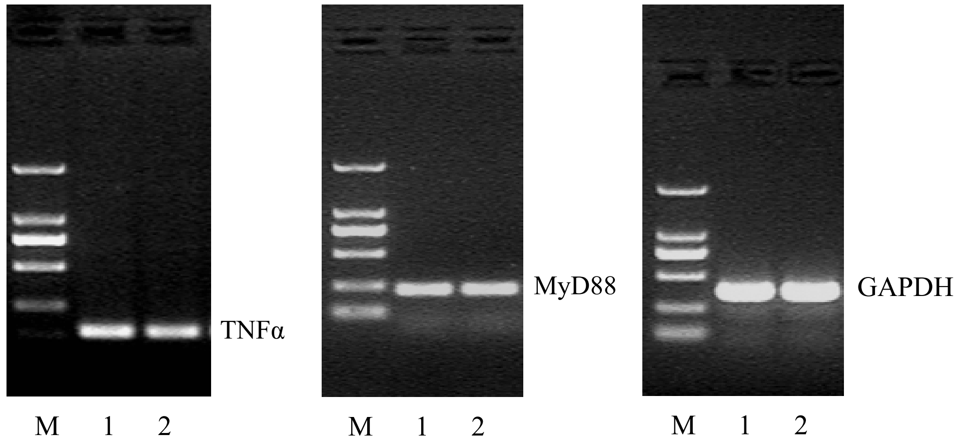

Figure 5. Full-length gels for ARIP2 mRNA in mouse peritoneal macrophages examined by RT-PCR in manuscript Figure 6A. M, molecular weight (bp); lane1, control pcDNA3 empty plasmids; lane2, pcDNA3-ARIP2 expressing plasmids.

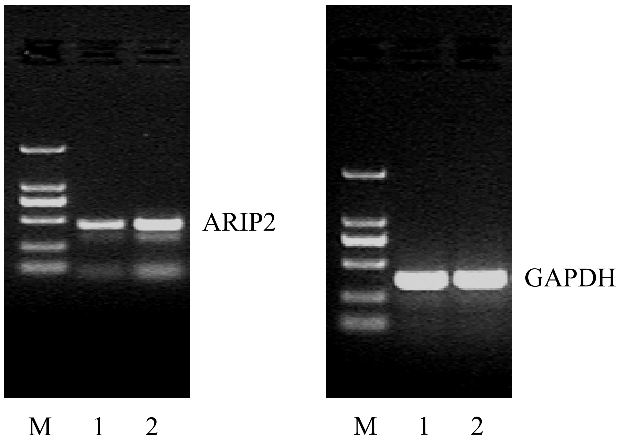

Supplement: Supplementary file 1 — Supplementary Information of full-length gels [file 41598_2017_10855_MOESM1_ESM.pdf]
